# Computed Tomography Severity Index vs. Other Indices in the Prediction of Severity and Mortality in Acute Pancreatitis: A Predictive Accuracy Meta-analysis

**DOI:** 10.3389/fphys.2019.01002

**Published:** 2019-08-27

**Authors:** Alexandra Mikó, Éva Vigh, Péter Mátrai, Alexandra Soós, András Garami, Márta Balaskó, László Czakó, Bernadett Mosdósi, Patrícia Sarlós, Bálint Erőss, Judit Tenk, Ildikó Rostás, Péter Hegyi

**Affiliations:** ^1^Institute for Translational Medicine, Medical School, University of Pécs, Pécs, Hungary; ^2^Department of Radiology, Medical School, University of Pécs, Pécs, Hungary; ^3^Department of Central Radiology, Markusovszky University Teaching Hospital, Szombathely, Hungary; ^4^Clinical Medicine Doctoral School, University of Szeged, Szeged, Hungary; ^5^Institute of Bioanalysis, Medical School, University of Pécs, Pécs, Hungary; ^6^Department of Pathophysiology, University of Szeged, Szeged, Hungary; ^7^Department of Paediatrics, Medical School, University of Pécs, Pécs, Hungary; ^8^Division of Gastroenterology, First Department of Medicine, Medical School, University of Pécs, Pécs, Hungary; ^9^Division of Translational Medicine, First Department of Medicine, University of Pécs, Pécs, Hungary; ^10^Hungarian Academy of Sciences, University of Szeged Momentum Gastroenterology Multidisciplinary Research Group, Szeged, Hungary

**Keywords:** acute pancreatitis, CT-severity index, accuracy, severity, mortality

## Abstract

**Background:** The management of the moderate and severe forms of acute pancreatitis (AP) with necrosis and multiorgan failure remains a challenge. To predict the severity and mortality of AP multiple clinical, laboratory-, and imaging-based scoring systems are available.

**Aim:** To investigate, if the computed tomography severity index (CTSI) can predict the outcomes of AP better than other scoring systems.

**Methods:** A systematic search was performed in three databases: Pubmed, Embase, and the Cochrane Library. Eligible records provided data from consecutive AP cases and used CTSI or modified CTSI (mCTSI) alone or in combination with other prognostic scores [Ranson, bedside index of severity in acute pancreatitis (BISAP), Acute Physiology, and Chronic Health Examination II (APACHE II), C-reactive protein (CRP)] for the evaluation of severity or mortality of AP. Area under the curves (AUCs) with 95% confidence intervals (CIs) were calculated and aggregated with STATA 14 software using the metandi module.

**Results:** Altogether, 30 studies were included in our meta-analysis, which contained the data of 5,988 AP cases. The pooled AUC for the prediction of mortality was 0.79 (CI 0.73–0.86) for CTSI; 0.87 (CI 0.83–0.90) for BISAP; 0.80 (CI 0.72–0.89) for mCTSI; 0.73 (CI 0.66–0.81) for CRP level; 0.87 (CI 0.81–0.92) for the Ranson score; and 0.91 (CI 0.88–0.93) for the APACHE II score. The APACHE II scoring system had significantly higher predictive value for mortality than CTSI and CRP (*p* = 0.001 and *p* < 0.001, respectively), while the predictive value of CTSI was not statistically different from that of BISAP, mCTSI, CRP, or Ranson criteria. The AUC for the prediction of severity of AP were 0.80 (CI 0.76–0.85) for CTSI; 0.79, (CI 0.72–0.86) for BISAP; 0.83 (CI 0.75–0.91) for mCTSI; 0.73 (CI 0.64–0.83) for CRP level; 0.81 (CI 0.75–0.87) for Ranson score and 0.80 (CI 0.77–0.83) for APACHE II score. Regarding severity, all tools performed equally.

**Conclusion:** Though APACHE II is the most accurate predictor of mortality, CTSI is a good predictor of both mortality and AP severity. When the CT scan has been performed, CTSI is an easily calculable and informative tool, which should be used more often in routine clinical practice.

## Introduction

### Rationale

Acute pancreatitis (AP) is an inflammatory disease of the pancreas, one of the most common causes of hospitalization among gastrointestinal diseases (Lankisch et al., 2015). Based on the revised Atlanta classification, the severity of AP may be mild, moderate, or severe (Banks et al., 2013). Most cases of AP are mild, but the management of the moderate and severe forms of the disease with necrosis and multiorgan failure remains a challenge. The prognosis of the severe form is poor, it occurs in 8.8% of AP (Parniczky et al., 2016) and the mortality of severe AP (SAP) may reach 28% (Parniczky et al., 2016). Therefore, it is necessary to predict the severity of the disease because the early escalation of care and aggressive therapy may prevent complications and adverse outcomes of AP in high-risk patients. Unfortunately, research on pancreatitis is in danger, therefore, attempts to obtain clinically relevant data has very high importance (Szentesi et al., 2016).

### Objectives

Currently, there are various scoring systems used for the early prediction of SAP. First, the Ranson score was used (Ranson and Pasternack, 1977), but later the acute physiology and chronic health examination II (APACHE II) scoring system seemed to be more accurate (Yeung et al., 2006). Moreover, several inflammatory parameters such as C-reactive protein (CRP) and interleukin-6 were documented to be clinically relevant in the differentiation of mild and non-mild AP (Sternby et al., 2017). In 2008, a new, easy-to-implement bedside index of severity in acute pancreatitis (BISAP) was proposed for use within 24 h of hospitalization to predict in-hospital mortality (Wu et al., 2008). However, with the improvements of the imaging techniques, contrast-enhanced computed tomography (CT) gets an important place in the diagnosis of AP and its complications. In the early ‘90s, Balthazar and his coworkers developed a numerical scoring system, the CT severity index (CTSI), for the estimation of the severity of AP (Balthazar et al., 1990). It combines the quantification of pancreatic and peripancreatic inflammation with the extent of pancreatic parenchymal necrosis. In 2004, Mortele et al., formulated the modified CTSI (mCTSI) including a simplified evaluation of peripancreatic inflammation and extent of pancreatic parenchymal necrosis and incorporated the extrapancreatic complications (vascular, gastrointestinal, and extrapancreatic parenchymal complications as well as the presence of pleural effusion and/or ascites) in the assessment. This modified index correlated more with the outcome of AP (Mortele et al., 2004). Tables 1A,B show the components of CTSI and mCTSI.

**Table 1:** Components of the CTSI and mCTSI.

**(A) Components of the CTSI**	
Pancreatic inflammation	
Normal pancreas	0
Focal or diffuse enlargement of the pancreas	1
Intrinsic pancreatic abnormalities with inflammatory changes in peripancreatic fat	2
Single, ill-defined fluid collection or phlegmon	3
Two or more poorly defined collections or presence of gas in or adjacent to the pancreas	4
Pancreatic necrosis	
None	0
≤30%	2
>30% and ≤ 50%	4
>50%	6
**(B) Components of the mCTSI**	
Pancreatic inflammation	
Normal pancreas	0
Intrinsic pancreatic abnormalities with peripancreatic inflammatory changes	2
Pancreatic or peripancreatic fluid collection or peripancreatic fat necrosis	4
Pancreatic necrosis	
None	0
Less 30%	2
>30%	4
Extrapancreatic complications	
Pleural effusion, ascites, vascular complication (venous thrombosis, arterial hemorrhage, pseudoaneurysm), parenchymal complication (infarction, hemorrhage, subcapsular fluid collection), GI involvement (inflammation, perforation, intraluminal fluid collection)	2

### Research Question

It is still not clear, which scoring system has the highest predictive accuracy for severity and mortality of AP. The aim of this meta-analysis was to investigate, how accurate CT-based severity indices are in the prediction of the severity and mortality of AP in comparison with other widely accepted and used scoring systems.

## Methods

### Study Design, Participants, Interventions, Comparators

The systematic search was conducted according to the Preferred Reporting Items for Systematic Reviews and Meta-Analyses (PRISMA) Statement (Moher et al., 2009) (Supplementary Document 1). We included studies on AP that used CTSI or mCTSI (index test) and compared them to any of the other scoring systems (Ranson, BISAP, APACHE II) or CRP (reference standards) in terms of their predictive value.

### Search Strategy

A systematic search was performed in Pubmed, Embase, and the Cochrane Library (CENTRAL), using the following search query: “*acute pancreatitis” AND (“computed tomography severity index” OR CTSI OR “modified computed tomography severity index” OR MCTSI)*. English language filter was used. Search key in Embase were: *(‘acute pancreatitis’/exp OR ‘acute pancreatitis’) AND (ctsi OR mctsi OR ‘computed tomography severity index’/exp OR ‘computed tomography severity index’ OR ‘modified computed tomography severity index’) AND [english]/lim*. The search was conducted on 11th March 2018. Duplicates were removed using the (EndNote X7.4, Clarivate Analytics, Philadelphia, PA, US) reference manager software.

### Study Selection and Data Extraction

Two independent investigators (EV, AM) selected the studies, and disagreements were resolved by a third reviewer (PH). The records were selected for meta-analysis if (1) AP patients of any severity were enrolled consecutively; (2) if CTSI or mCTSI were used for the prediction of the severity or mortality of AP; and (3) if sensitivity and specificity values, the absolute numbers of true positive (TP), false negative (FN), false positive (FP) and true negative (TN), and/or area under the curve (AUC) were reported (for CTSI/mCTSI regarding AP severity and/or mortality). If other prognostic scores (Ranson, BISAP, APACHE II) or CRP values were also assessed in the selected articles, those results were extracted as well. Only full-text articles were included.

Studies, which met the inclusion criteria were assessed for full-text evaluation. The following data were extracted from the articles: first author; year of the publication; study period; study design; the AP scoring systems used; evaluation time of the scores; the used definition for SAP; sample size based on severity; mean age; male/female ratio; cut-off value, clinical end-points, and several data about the statistical analysis were reviewed for the risk of bias assessment.

### Data Analysis

To construct 2 × 2 contingency tables, true positive, false positive, false negative, and true negative values were abstracted. These served as input to fit Hierarchial Summary Receiver Operating Characteristics (HSROC) curves and estimate summary sensitivity, specificity, and diagnostic odds ratio (DOR) with 95% confidence intervals (CI). For each method and outcome, we collected the AUC values and their CIs as well and performed a meta-analysis using the random effect model to gain pooled AUC estimates with 95% CI.

The statistical analysis was performed with Stata 14 software using the metandi module^1^. Heterogeneity was assessed using the I^2^ measure and the corresponding chi^2^ test, *p* < 0.1 indicates significant heterogeneity. Based on the Cochrane Handbook, I^2^ = 100% × (Q–df)/Q represents the magnitude of the heterogeneity (moderate: 30–60%, substantial: 50–90%, considerable: 75–100%) (Higgins and Green, 2011).

### Risk of Bias and Applicability Assessment

The Prediction model Risk Of Bias ASsessment Tool (PROBAST) (Wolff et al., 2019) was used to assess the risk of bias and applicability of primary studies in accordance with the recommendation of the Cochrane Collaboration. This tool is able to assess the risk of bias based on the following four domains: participants, predictors, outcome, and analysis. It includes also concerns regarding applicability in three domains: participants, predictors and outcome.

## Results

### Study Selection and Characteristics

**Figure 1:**
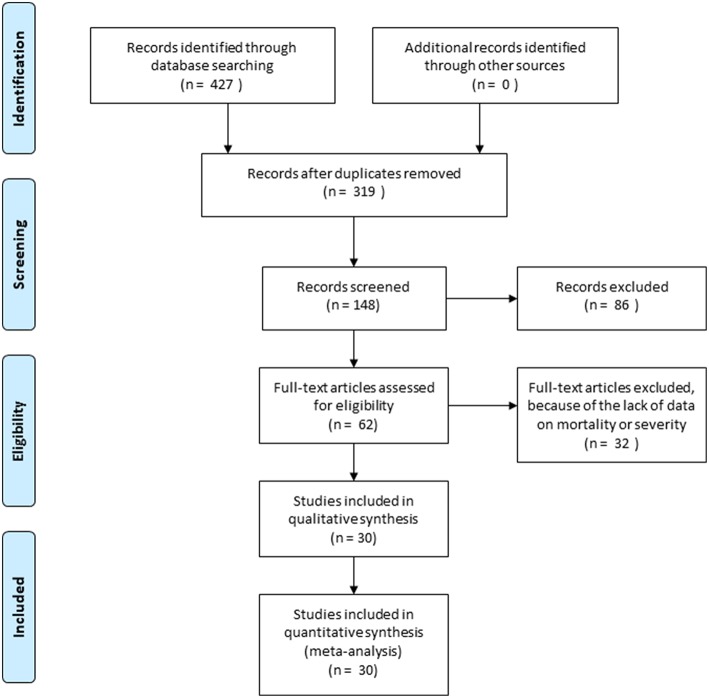
Flowchart of study selection.

The systematic search strategy identified 319 articles after removal of duplicates. The search provided 123 articles in Pubmed, 279 studies in Embase and 25 articles in Cochrane library databases. The flow chart (Figure 1) shows the study selection process: 148 articles were screened by abstract and after excluding 86 studies, 62 full-text articles were assessed for eligibility. A further 32 articles were excluded, because of the lack of reporting of the outcome of interest or inappropriate patient population. In our meta-analysis 30 articles were included (Simchuk et al., 2000; Chatzicostas et al., 2003; Gurleyik et al., 2005; Vriens et al., 2005; Huang et al., 2010; Papachristou et al., 2010; Bollen et al., 2011, 2012; Fabre et al., 2012; Lautz et al., 2012; Jakchairoongruang and Arjhansiri, 2013; Khanna et al., 2013; Park et al., 2013; Xu et al., 2014; Banday et al., 2015; Cho et al., 2015; Qiu et al., 2015; Sharma et al., 2015; Yue et al., 2015; Alper et al., 2016; Hashimoto et al., 2016; Lee et al., 2016; Raghuwanshi et al., 2016; Yadav et al., 2016; Yang et al., 2016; Zhao et al., 2016; Biberoglu et al., 2017; Kumar et al., 2017; Sahu et al., 2017; Fei et al., 2018), which contained the data of 5,988 patients. The main characteristics of the included studies are shown in Table 2.

**Table 2:** Basic characteristics of the included studies.

**Study number**	**References**	**Study period**	**Study design**	**Country**	**Groups**	**Evaluation time, h**	**Definition of SAP**	**Sample size**	**Age**	**Male/female ratio**	**Severity**	**Mortality**
1	Cho et al., 2015	2011–2012	Prospective	South Korea	CTSI	<48 of admission	2012 revised Atlanta	161	62.3^a^ ± 16.1^c^	102/59	+	
					Ranson	<48 of admission						
					BISAP	<24 of admission						
					APACHE II	<24 of admission						
					CRP	On admission and 24 h after admission						
2	Gurleyik et al., 2005	2001–2005	Prospective	Turkey	CTSI	<120 after the onset of symptoms	1992 Atlanta	55	Mild: 56.1^a^ (28–98)^e^ severe: 60.3^a^ (23–77)^e^	18/37	+	
					APACHE II	48						
					CRP	at 48 h						
3	Lee et al., 2016	2010–2013	Prospective	South Korea	CTSI	On admission	2012 revised Atlanta	146	Mild: 48.8^a^ ± 16.4^c^, moderate: 49.3^a^ ± 18.5^c^, severe: 63^a^ ± 22.8^c^	92/54	+	
					Ranson	On admission						
					BISAP	On admission						
					APACHE II	On admission						
					CRP	On admission and 24 h after admission						
4	Qiu et al., 2015	2008–2014	Retrospective	China	CTSI	<48 h of admission	2012 revised Atlanta	129	57^a^ ± 17.3^c^	468/441	+	
					Ranson	<48 h of admission						
					BISAP	<24 h of admission						
					CTSI	<48 h of admission		780				
					Ranson	<48 h of admission						
					BISAP	<24 h of admission						
5	Yue et al., 2015	2011–2013	Prospective	China	CTSI	<48 h of admission	2009 Atlanta	169	54.3^a^ ± 16.7^c^	98/71	+	
					Ranson	<48 h						
					APACHE II	0., and 72 h						
6	Biberoglu et al., 2017	2010–2011	Retropsective	Turkey	CTSI	NA	NA	76	Presented in intervals	29/47		+
7	Bollen et al., 2011	2005–2007	Prospective	NA	CTSI	0–168 h, median 48 h after onset of symptoms	2009 Atlanta	196	53^a^ (21–94)^e^	107/89	+	+
					MCTSI	0–168 h, median 48 h after onset of symptoms						
					APACHE II	NA						
8	Sharma et al., 2015	2013–2014	Retropsective	India	CTSI	72–240 h after onset of symptoms	2012 revised Atlanta	105	40.6^a^ ± 12.99^NA^	65/40		+
					MCTSI	72–240 h after onset of symptoms						
					BISAP	NA						
9	Yang et al., 2016	2007–2015	Retropsective	China	MCTSI	<72 after onset of symptoms	2008 Atlanta	326	44^a^ (14–85)^e^	184/142	+	+
					BISAP	<24 of admission						
					Ranson	<48 of admission						
					APACHE II	<24 of admission						
10	Bollen et al., 2012	2.5 year period	Prospective	USA	CTSI	<24 of admission	2009 Atlanta	131	54^b^ (21–91)^e^	84/66	+	+
					MCTSI	<24 of admission		131				
					APACHE II	NA		131/159				
					BISAP	NA		131/159				
11	Alper et al., 2016	2011–2014	Prospective	Turkey	CTSI	Within 72–96 h after admission	CTSI >6 and/or modified Glasgow score >3	187	mild 57^a^ ± 17.9^NA^, severe 60.1^a^ ± 15.9^NA^	111/76	+	+
12	Zhao et al., 2016	2012–2014	Observational	China	APACHE II	At admission = <72 h after onset of symptoms	2012 revised Atlanta	74	NA	NA	+	
					CTSI	At admission = <72 h after onset of symptoms						
					CRP	<72 h						
13	Fabre et al., 2012	2003–2007	Retrospective	France	Ranson	NA	1992 Atlanta	48	133 months^b^ (24.9–233.5)^e^	23/25	+	
					CTSI			17				
14	Simchuk et al., 2000	1992–1997	Retrospective	USA	CTSI	–	–	268	57^a^ (18–93)^e^	147/121		+
15	Jakchairoongruang and Arjhansiri, 2013	2005–2010	Retrospective	Thailand	CTSI	–	–	72	47.7^a^ (6–89)^e^	39/33		+
16	Hashimoto et al., 2016	2002–2012	Retrospective	Japan	Ranson	NA	DeBanto et al.	37	6^b^ (5–12)^e^	15/22	+	
					CTSI			33				
17	Park et al., 2013	2007–2010	Retrospective	Korea	BISAP	<24 of admission	Based on organ failure and/or local complications	303	52^NA^ ± 17^NA^	216/87	+	+
					Ranson	<48 of admission						
					APACHE II	<24 of admission						
					CTSI	Within 7 days of admission						
					CRP	Initial						
					CRP	After 48 h						
18	Khanna et al., 2013	2010–2012	Prospective	India	BISAP	<24 of admission	Presence of organ failure for more than 48 h and local complications	72	40.5^a^ (18–76)^e^	37/35	+	+
					APACHE II	<24 of admission		72				
					Ranson	<48 of admission		72				
					CTSI	On day 4		54				
					CRP	On day 2		60				
19	Lautz et al., 2012	2000–2009	Retrospective	USA	CTSI	At presentation	DeBanto et al.	64	12.3^b^	NA	+	
					Ranson	NA		64				
20	Chatzicostas et al., 2003	1999–2001	Prospective	Greece	CTSI	<72 h after admission (median time 62 h)	1992 Atlanta	78	63.8^a^ (25–93)^e^	42/36	+	
					APACHE II	<24 of admission						
					Ranson	<48 of admission						
21	Xu et al., 2014	2012	Retropsective	China	CTSI	Within 3–5 days after admission	2008 Atlanta	257	51.2^a^ ± 12.3^NA^ (23–89)^e^	196/61	+	
22	Yadav et al., 2016	2012–2014	Prospective	India	BISAP	<24 of admission	Persistent organ failure for more than 48 h	119	38.94^a^ ± 14.59^c^	84/35	+	+
					Ranson	<48 of admission						
					CTSI	Within the first 7 days of hospitalization						
23	Papachristou et al., 2010	2003–2007	Prospective	USA	BISAP	<24 of admission	Presence of organ failure for more than 48 h	185	51.7^a^(15–90)^e^	94/91	+	+
					Ranson	<48 of admission						
					APACHE II	<24 of admission						
					CTSI	Within 48 h from admission						
24	Vriens et al., 2005	1994–2002	Prospective	Netherlands	CTSI	Within 48 h after admission, 80% within 12 h	NA	79	61^a^ (15–86)^e^	39/40		+
					Ranson	<48 of admission						
25	Raghuwanshi et al., 2016	2013–2015	Prospective	India	CTSI	NA	2012 revised Atlanta	50	NA	NA		+
					MCTSI							
26	Banday et al., 2015	2012–2013	Prospective	India	MCTSI	NA	NA	50	42,32^a^ (17–80)^e^	33/17		+
27	Huang et al., 2010	2007–2009	Prospective	China	CTSI	<24 h after onset of symptoms	1992 Atlanta	187	mild: 60.4^a^ ± 6.7^c^ severe: 59.5^a^ ± 7.1^c^,	112/75	+	
28/A	Fei et al., 2018	2013–2016	Retrospective	China	BISAP	On admission	2012 revised Atlanta	1073	47.3^a^ ± 7.1^c^	615/458	+	
					CTSI							
					AAPCHE II							
					Ranson							
28/B		2012–2016			BISAP			326	53.6^a^ ± 8.7^c^	168/158		
					CTSI							
					APACHE II							
					Ranson							
29	Kumar et al., 2017	2014–2016	Prospective	Nepal	CTSI	After 48 h after arrival to hospital	Atlanta	125	46.78^a^ ± 14.16^c^	74/51	+	
					CRP	at 48 h						
					Ranson	After 48 h of admission						
30	Sahu et al., 2017	2014–2016	Prospective	India	CTSI	Median of 6 days; range of 5–11 days	2012 revised Atlanta	60	37^a^ (19–65)^e^	36/24		+
					mCTSI	Median of 6 days; range of 5–11 days						

CTSI, computed tomography severity index; MCTSI, modified computed tomography severity index; BISAP, bedside index of severity in acute pancreatitis; APACHE II, Acute Physiology And Chronic Health Examination II; N/A, not applicable;

a mean;

b median;

c standard difference;

d standard error of mean;

e *range*.

### Prediction of Mortality

**Figure 2:**
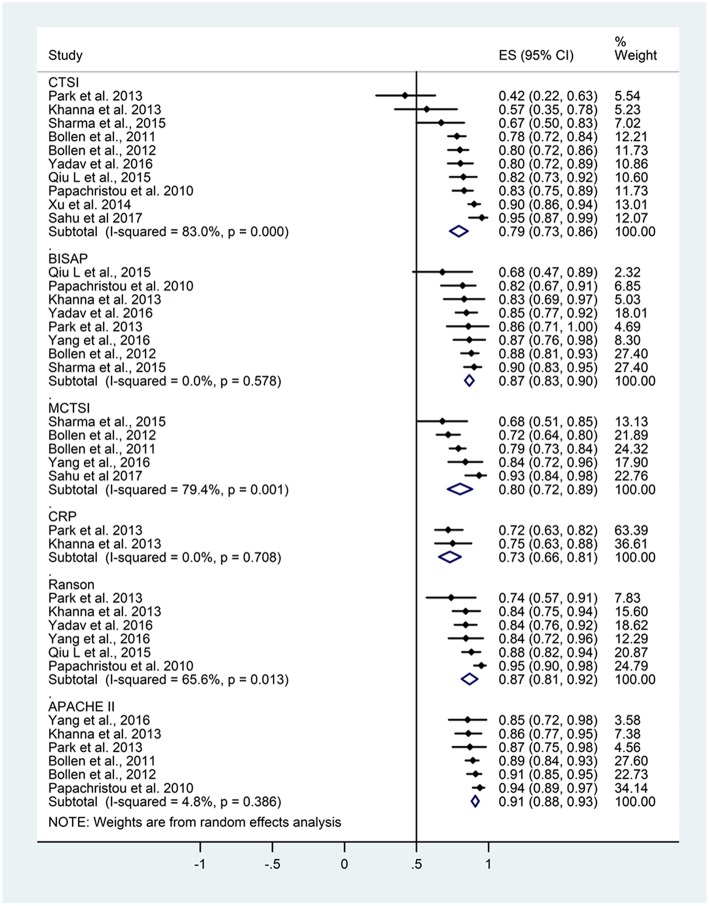
Area under the curve (AUC) summarizing the predictive performance of scoring systems regarding mortality in acute pancreatitis. Size of squares for effect size reflects weight of studies in pooled analysis. Horizontal bars represent 95% confidence intervals (CI). CTSI, computed tomography severity index; BISAP, bedside index of severity in acute pancreatitis; mCTSI, modified computed tomography severity index; CRP, C-reactive protein; APACHE II, Acute Physiology And Chronic Health Examination II. The vertical line represents the line of no effect.

From the 30 articles, 11 contained data on AUC for the prediction of mortality (Figure 2). Table 3 summarizes the study numbers, sample sizes, AUC, and heterogeneity data of the different severity scores based on the outcome of AP. For CTSI based on data from 10 articles, the pooled AUC for mortality was 0.79 (CI 0.73–0.86; heterogeneity *I*^2^ = 83%, *p* < 0.001). Eight articles included AUC data for mortality for BISAP, the pooled AUC was 0.87 (CI 0.83–0.90; heterogeneity *I*^2^ = 0%, *p* = 0.578). The pooled AUC for mCTSI was 0.80 (CI 0.72–0.89; heterogeneity I^2^ = 79.4%, *p* = 0.001) according to five studies. Only two studies reported AUC data predicting mortality for CRP level, and the pooled AUC was 0.73 (CI 0.66–0.81; heterogeneity *I*^2^ = 0%, *p* = 0.708) Six articles included AUC data for mortality for Ranson score with a pooled AUC of 0.87 (CI 0.81–0.92; heterogeneity *I*^2^ = 65.6%, *p* = 0.013) and also for APACHE II score with a pooled AUC of 0.91 (CI 0.88–0.93; heterogeneity *I*^2^ = 4.8%, *p* = 0.386).

**Table 3:** Summary table of mortality and severity data based on the forest plots.

**Score**	**Study number**	**Sample size**	**AUC (CI)**	**Heterogeneity**
**MORTALITY**				
CTSI	10	1,489	0.79 (0.73–0.86)	*I*^2^ = 83%, *p* < 0.001
BISAP	8	1,370	0.87 (0.83–0.90)	*I*^2^ = 0%, *p* = 0.578
mCTSI	5	818	0.80 (0.72–0.89)	*I*^2^ = 79.4%, *p* = 0.001
CRP	2	363	0.73 (0.66–0.81)	*I*^2^ = 0%, *p* = 0.708
Ranson	6	1,134	0.87 (0.81–0.92)	*I*^2^ = 65.6%, *p* = 0.013
APACHE II	6	1,213	0.91 (0.88–0.93)	*I*^2^ = 4.8%, *p* = 0.386
**SEVERITY**				
CTSI	18	2,535	0.80 (0.76–0.85)	*I*^2^ = 86.2%; *p* < 0.001
BISAP	10	1,898	0.79 (0.72–0.86)	*I*^2^ = 89.7%, *p* < 0.001
mCTSI	3	653	0.83 (0.75–0.91)	*I*^2^ = 68.1%; *p* = 0.043
CRP	6	869	0.73 (0.64–0.83)	*I*^2^ = 77%, *p* = 0.001
Ranson	14	2,119	0.81 (0.75–0.87)	*I*^2^ = 87.5%, *p* < 0.001
APACHE II	11	1,198	0.80 (0.77–0.83)	*I*^2^ = 36.8%, *p* = 0.105

Based on the above results of the meta-analytical calculations the APACHE II scoring system had significantly higher predictive accuracy for mortality than CTSI or CRP level (*p* = 0.001; *p* < 0.001, respectively). However, CTSI was not different from the BISAP, mCTSI, CRP or Ranson criteria in the prediction of mortality of AP, and these scores can be classified as good and fair.

### Prediction of Severity

**Figure 3:**
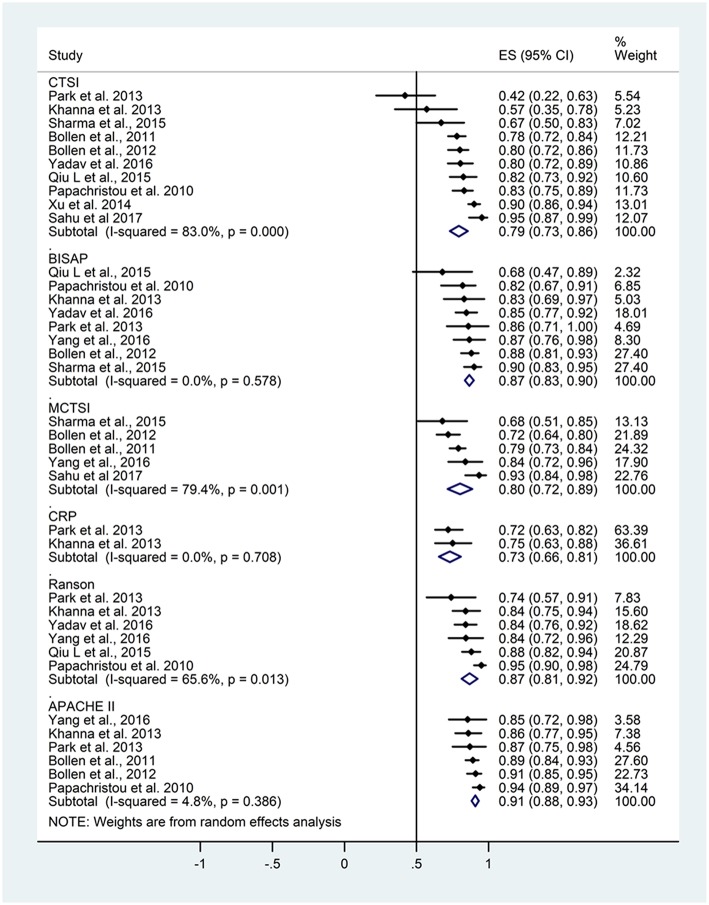
Area under the curve (AUC) summarizing the predictive performance of scoring systems regarding severity in acute pancreatitis. Size of squares for effect size reflects weight of trial in pooled analysis. Horizontal bars represent 95% confidence intervals (CI). CTSI, computed tomography severity index; BISAP, bedside index of severity in acute pancreatitis; mCTSI, modified computed tomography severity index; CRP, C-reactive protein; APACHE II, Acute Physiology And Chronic Health Examination II. The vertical line represents the line of no effect.

AUC data for severity were included in 19 studies (Figure 3).

The pooled AUC for severity based on data from 18 articles was 0.80 (CI 0.76–0.85) for CTSI with high heterogeneity (I^2^ = 86.2%; *p* < 0.001). The pooled AUC for BISAP was 0.79 (CI 0.72–0.86, heterogeneity *I*^2^ = 89.7%, *p* < 0.001). The pooled AUC for mCTSI from three studies was 0.83 (CI 0.75–0.91) with substantial heterogeneity (*I*^2^ = 68.1%; *p* = 0.043). The pooled AUC was 0.73 (CI 0.64–0.83) for CRP level (heterogeneity: *I*^2^ = 77%, *p* = 0.001), 0.81 (CI 0.75–0.87) for Ranson score (heterogeneity *I*^2^ = 87.5%, *p* < 0.001), and 0.80 (CI 0.77–0.83) for APACHE II score (heterogeneity *I*^2^ = 36.8%, *p* = 0.105).

There was no statistical difference between the severity predicting values of the different scoring systems. The heterogeneity values were *I*^2^ = 86.2%, *p* < 0.001; *I*^2^ = 89.7%, *p* < 0.001; *I*^2^ = 68.1%, *p* = 0.043; *I*^2^ = 77%, *p* = 0.001; *I*^2^ = 87.5%, *p* < 0.001; *I*^2^ = 36.8%, *p* = 0.105 for CTSI, BISAP, mCTSI, CRP, Ranson, and APACHE II scores, respectively. The heterogeneity across the studies was significant in all scoring systems or predicting values, except for the APACHE II score. Based on the results of the meta-analytical calculations, the severity prediction values of the included scoring systems are not different.

### HSROC Analysis of Different Scoring Systems for Predicting Mortality and Severity of AP

**Figure 4:**
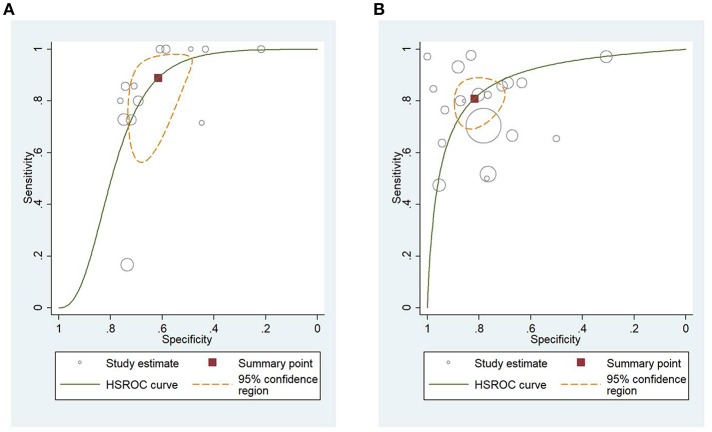
**(A)** Hierarchial summary receiver operating characteristic curves (HSROC) for computed tomography severity index (CTSI) for predicting mortality of acute pancreatitis. **(B)** HSROC for CTSI for predicting severity of acute pancreatitis.

**Figure 5:**
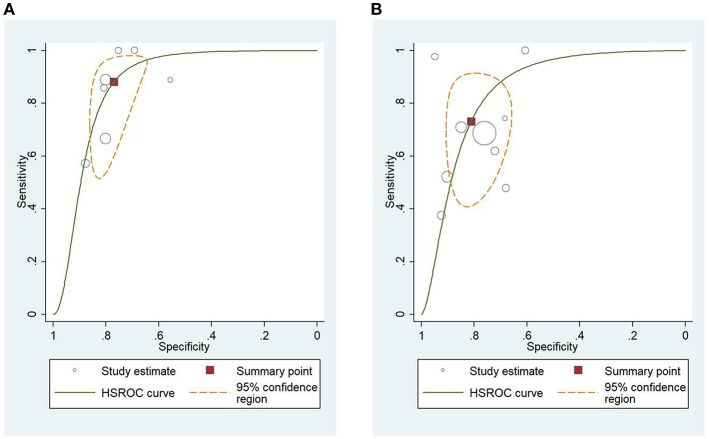
**(A)** Hierarchial summary receiver operating characteristic curves (HSROC) for bedside index of severity in acute pancreatitis (BISAP) for predicting mortality. **(B)** HSROC for BISAP for predicting severity of acute pancreatitis.

**Figure 6:**
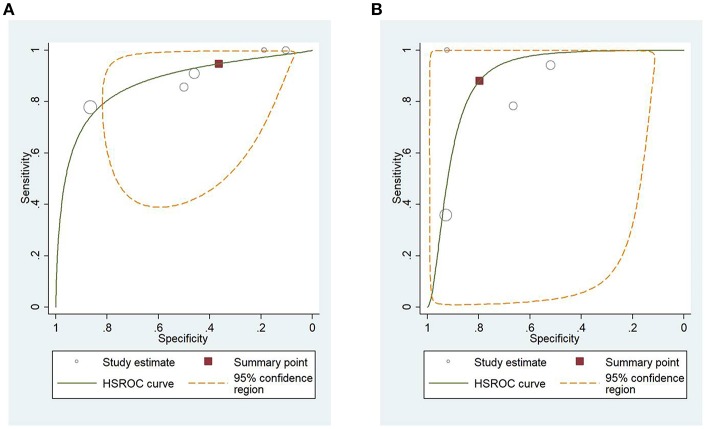
**(A)** Hierarchial summary receiver operating characteristic curves (HSROC) for modified computed tomography severity index (mCTSI) for predicting mortality of acute pancreatitis. **(B)** HSROC for mCTSI for predicting severity of acute pancreatitis.

**Figure 7:**
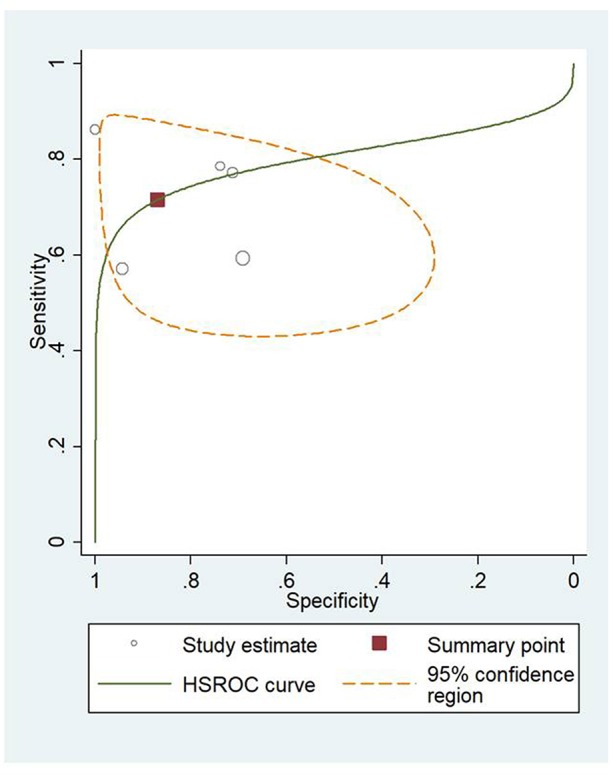
Hierarchial summary receiver operating characteristic curves (HSROC) for C-reactive protein (CRP) for predicting severity of acute pancreatitis.

**Figure 8:**
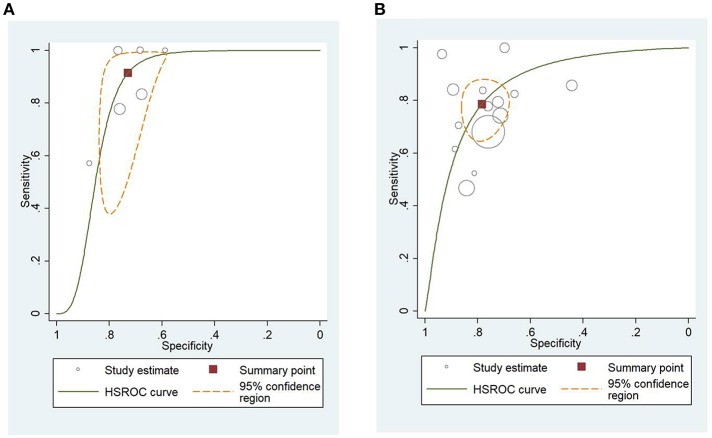
**(A)** Hierarchial summary receiver operating characteristic curves (HSROC) for Ranson score for predicting mortality of acute pancreatitis. **(B)** HSROC for Ranson score for predicting severity of acute pancreatitis.

**Figure 9:**
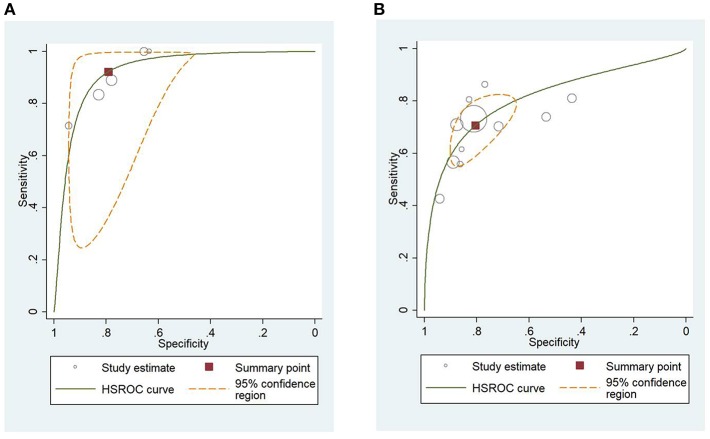
**(A)** Hierarchial summary receiver operating characteristic curves (HSROC) for Acute Physiology And Chronic Health Examination II (APACHE II) score for predicting mortality of acute pancreatitis. **(B)** HSROC for APACHE II score for predicting severity of acute pancreatitis.

Figure 4A shows the application of CTSI in the form of HSROC curves for mortality [sensitivity: 0.88 (CI 0.69–0.97); specificity: 0.61 (CI 0.52–0.70); DOR: 12.84 (CI 4.19–39.41)] and Figure 4B for severity, respectively [sensitivity: 0.81 (CI 0.73–0.87); specificity: 0.82 (CI 0.73–0.88); DOR: 19.1 (CI 10.29–35.45)]. Figure 5A illustrates the bivariate HSROC curve of BISAP score for mortality [sensitivity: 0.88 (CI 0.71–0.96); specificity: 0.77 (CI 0.70–0.83); DOR: 24.74 (CI 9.44–64.81)] and Figure 5B for severity, respectively [sensitivity: 0.73 (CI 0.53–0.87); specificity: 0.80 (CI 0.72–0.88); DOR: 11.71 (CI 4.49–30.61)]. Figure 6A shows the application of mCTSI in the form of HSROC curves for mortality [sensitivity: 0.95 (CI 0.76–0.99); specificity: 0.36 (CI 0.16–0.63); DOR: 10.32 (CI 2.11–50.53)] and Figure 6B for severity, respectively [sensitivity: 0.88 (CI 0.47–0.98); specificity: 0.80 (CI 0.56–0.92); DOR: 29.07 (CI 3.36–251.91)]. Figure 7 illustrates the bivariate HSROC curve of CRP score for severity [sensitivity: 0.71 (CI 0.59–0.81); specificity: 0.87 (CI 0.66–0.96); DOR: 16.75 (CI 3.49–80.48)]. Figure 8A illustrates the bivariate HSROC curve of Ranson score for mortality [sensitivity: 0.91 (CI 0.70–0.98); specificity: 0.72 (CI 0.66–0.79); DOR: 28.72 (CI 7.57–109.05)] and Figure 8B for severity, respectively [sensitivity: 0.79 (CI 0.69–0.86); specificity: 0.78 (CI 0.71–0.84); DOR: 13.32 (CI 7.33–24.24)]. Figure 9A illustrates the bivariate HSROC curve of APACHE II score for mortality [sensitivity: 0.92 (CI 0.70–0.98); specificity: 0.79 (CI 0.66–0.88); DOR: 45.08 (CI 11.4–178.2)] and Figure 9B for severity, respectively [sensitivity: 0.71 (CI 0.60–0.79); specificity: 0.80 (CI 0.71–0.88); DOR: 9.94 (CI 6.45–15.30)].

Sensitivity, specificity, and DOR data of all scores predicting mortality and severity are summarized in Table 4. In summary, the sensitivity for the prediction of mortality of AP was the highest for mCTSI, Ranson, and APACHE II scores. While the specificity for prediction of mortality was the best for APACHE II, BISAP, and Ranson scores. The sensitivity for the prediction of severity of CTSI, mCTSI, and Ranson scores were the highest, while the specificity for prediction of severity were the highest for CRP and CTSI.

**Table 4:** Summary table of mortality and severity data based on the HSROC curves.

**Score**	**Sensitivity**	**Specificity**	**DOR**
**MORTALITY**			
CTSI	0.88 (0.69–0.97)	0.61 (0.52–0.70)	12.84 (4.19–39.41)
BISAP	0.88 (0.71–0.96)	0.77 (0.70–0.83)	24.74 (9.44–64.81)
MCTSI	0.95 (0.76–0.99)	0.36 (0.16–0.63)	10.32 (2.11–50.53)
Ranson	0.91 (0.70–0.98)	0.72 (0.66–0.79)	28.72 (7.57–109.05)
APACHE II	0.92 (0.70–0.98)	0.79 (0.66–0.88)	45.08 (11.4–178.2)
**SEVERITY**			
CTSI	0.81 (0.73–0.87)	0.82 (0.73–0.88)	19.10 (10.29–35.45)
BISAP	0.73 (0.53–0.87)	0.80 (0.72–0.88)	11.71 (4.49–30.61)
MCTSI	0.88 (0.47–0.98)	0.80 (0.56–0.92)	29.07 (3.36–251.91)
CRP	0.71 (0.59–0.81)	0.87 (0.66–0.96)	16.75 (3.49–80.48)
Ranson	0.79 (0.69–0.86)	0.78 (0.71–0.84)	13.32 (7.33–24.24)
APACHE II	0.71 (0.60–0.79)	0.80 (0.71–0.88)	9.94 (6.45–15.30)

### Quality Assessment and Risk of Bias

In this study, the two main outcomes were mortality and severity of the disease, therefore, we included the data in two different tables. The majority of the studies included in our analysis met the predefined criteria of the definition of AP and contained all grades of severities, therefore the risk of bias regarding the included populations was deemed as low. The data on CTSI from all studies were significantly limited by the timeframe the CT was done from either the admission or the onset of the symptoms and the diagnosis of AP. Therefore, this is the main limitation of the applicability of our results on CTSI. The result of the risk of bias and applicability assessment is showed in Tables 5A,B.

**TABLE 5A:** The PROBAST Tool for mortality.

**Study**	**Risk of bias**							**Applicability concerns**										**Overall**	
	**Partici-pants**	**Predictors**						**Outcome mortality**	**Ana-lysis**	**Partici-pants**	**Predictors**						**Outcome mortality**	**Risk of bias**	**Appli-cability**
		**CTSI**	**BISAP**	**MCTSI**	**CRP**	**Ran-son**	**Apache II**				**CTSI**	**BISAP**	**MCTSI**	**CRP**	**Ran-son**	**Apache II**			
Biberoglu et al., 2017	**+**	**?**	N/A	N/A	N/A	N/A	N/A	**–**	**–**	**–**	**?**	N/A	N/A	N/A	N/A	N/A	**–**	**+**	**?**
Bollen et al., 2011	**–**	**–**	N/A	**–**	N/A	N/A	**?**	**–**	**–**	**–**	**+**	N/A	**+**	N/A	N/A	**?**	**–**	**–**	**+**
Sharma et al., 2015	**–**	**–**	**–**	**–**	N/A	N/A	N/A	N/A	**–**	**–**	**+**	**?**	**+**	N/A	N/A	N/A	N/A	**–**	**+**
Yang et al., 2016	**–**	N/A	**–**	**–**	N/A	**–**	**–**	**–**	**?**	**+**	N/A	**–**	**–**	N/A	**–**	**–**	**–**	**–**	**+**
Bollen et al., 2012	**–**	**–**	**–**	**–**	N/A	N/A	**–**	**–**	**–**	**–**	**+**	**–**	**–**	N/A	N/A	**–**	**–**	**–**	**+**
Alper et al., 2016	**+**	**–**	N/A	N/A	N/A	N/A	N/A	**–**	**–**	**+**	**–**	N/A	N/A	N/A	N/A	N/A	**–**	**+**	**–**
Simchuk et al., 2000	**–**	**?**	N/A	N/A	N/A	N/A	N/A	**–**	**–**	**–**	**?**	N/A	N/A	N/A	N/A	N/A	N/A	**?**	**?**
Jakchairoongruang and Arjhansiri, 2013	**–**	**?**	N/A	N/A	N/A	N/A	N/A	**–**	**+**	**+**	**?**	N/A	N/A	N/A	N/A	N/A	**–**	**+**	**+**
Park et al., 2013	**–**	**–**	**–**	N/A	**–**	**–**	**–**	**–**	**–**	**–**	**+**	**–**	N/A	**–**	**–**	**–**	**–**	**–**	**+**
Khanna et al., 2013	**–**	**+**	**–**	N/A	**+**	**–**	**–**	**–**	**–**	**–**	**–**	N/A	N/A	**–**	**–**	**–**	**–**	**+**	**–**
Yadav et al., 2016	**+**	**–**	**–**	N/A	N/A	**–**	N/A	**–**	**–**	**–**	**+**	**–**	N/A	N/A	**–**	N/A	**–**	**+**	**+**
Papachristou et al., 2010	**–**	**+**	**–**	N/A	N/A	**–**	**–**	**–**	**–**	**–**	**+**	**–**	N/A	N/A	**–**	**–**	**–**	**+**	**+**
Vriens et al., 2005	**–**	**–**	N/A	N/A	N/A	**–**	N/A	N/A	**+**	**–**	**+**	N/A	N/A	N/A	**–**	N/A	**–**	**–**	**+**
Raghuwanshi et al., 2016	**+**	**?**	N/A	**?**	N/A	N/A	N/A	**–**	**?**	**?**	**+**	N/A	**+**	N/A	N/A	N/A	**–**	**+**	**+**
Banday et al., 2015	**–**	N/A	N/A	**?**	N/A	N/A	N/A	**–**	**?**	**–**	N/A	N/A	**+**	N/A	N/A	N/A	**–**	**?**	**+**
Sahu et al., 2017	**–**	**–**	N/A	**–**	N/A	N/A	N/A	**–**	**?**	**–**	**+**	N/A	**+**	N/A	N/A	N/A	**–**	**?**	**+**

**TABLE 5B:** The PROBAST Tool for severity.

**Study**	**Risk of bias**							**Applicability concerns**										**Overall**	
	**Partici-pants**	**Predictors**						**Outcome severity**	**Ana-lysis**	**Partici-pants**	**Predictors**						**Outcome severity**	**Risk of bias**	**Appli-cability**
		**CTSI**	**BISAP**	**MCTSI**	**CRP**	**Ran-son**	**Apache II**				**CTSI**	**BISAP**	**MCTSI**	**CRP**	**Ran-son**	**Apache II**			
Cho et al., 2015	**–**	**–**	**–**	N/A	**–**	**–**	**–**	**–**	**–**	**–**	**–**	**–**	N/A	**–**	**–**	**–**	**–**	**–**	**–**
Gurleyik et al., 2005	**–**	**–**	N/A	N/A	**–**	N/A	**–**	**–**	**–**	**–**	**+**	N/A	N/A	**–**	N/A	**–**	**–**	**–**	**+**
Lee et al., 2016	**–**	**–**	**–**	N/A	**–**	**–**	**–**	**–**	**?**	**–**	**+**	**–**	N/A	**–**	**–**	**–**	**–**	**?**	**+**
Qiu et al., 2015	**–**	**–**	**–**	N/A	N/A	**–**	N/A	**–**	**–**	**–**	**–**	**–**	N/A	N/A	**–**	N/A	**–**	**–**	**–**
Yue et al., 2015	**–**	**+**	N/A	N/A	N/A	**–**	**–**	**–**	**–**	**–**	**+**	N/A	N/A	N/A	**–**	**–**	**–**	**+**	**+**
Bollen et al., 2011	**–**	**–**	N/A	**–**	N/A	N/A	**?**	**–**	**–**	**–**	**+**	N/A	**+**	N/A	N/A	**?**	**–**	**–**	**+**
Yang et al., 2016	**–**	N/A	**–**	**–**	N/A	**–**	**–**	**–**	**?**	**+**	N/A	**–**	**–**	N/A	**–**	**–**	**–**	**–**	**+**
Bollen et al., 2012	**–**	**–**	**–**	**–**	N/A	N/A	**–**	**–**	**–**	**–**	**+**	**–**	**–**	N/A	N/A	**–**	**–**	**–**	**+**
Alper et al., 2016	**–**	**–**	N/A	N/A	N/A	N/A	N/A	**+**	**–**	**+**	**–**	N/A	N/A	N/A	N/A	N/A	**–**	**+**	**+**
Zhao et al., 2016	**–**	**–**	N/A	N/A	**–**	N/A	**–**	**–**	**–**	**+**	**–**	N/A	N/A	**–**	N/A	**–**	**–**	**–**	**+**
Fabre et al., 2012	**–**	**+**	N/A	N/A	N/A	**–**	N/A	**–**	**?**	**+**	N/A	N/A	N/A	N/A	**?**	N/A	**–**	**+**	**+**
Hashimoto et al., 2016	**–**	**?**	N/A	N/A	N/A	**?**	N/A	N/A	**+**	**+**	**?**	N/A	N/A	N/A	**?**	N/A	**?**	**+**	**+**
Park et al., 2013	**–**	**–**	**–**	N/A	**–**	**–**	**–**	**–**	**–**	**–**	**+**	**–**	N/A	**–**	**–**	**–**	**–**	**–**	**+**
Khanna et al., 2013	**–**	**+**	**–**	N/A	**+**	**–**	**–**	**–**	**–**	**–**	**–**	N/A	N/A	**–**	**–**	**–**	**–**	+	**–**
Lautz et al., 2012	**+**	**–**	N/A	N/A	N/A	**?**	N/A	**–**	**?**	**+**	**+**	N/A	N/A	N/A	**?**	N/A	N/A	+	**+**
Chatzicostas et al., 2003	**–**	**–**	N/A	N/A	N/A	**–**	**–**	**–**	**?**	**–**	**–**	N/A	N/A	N/A	**–**	**–**	**–**	**–**	**–**
Xu et al., 2014	**+**	**–**	N/A	N/A	N/A	N/A	N/A	**–**	**–**	**–**	**–**	N/A	N/A	N/A	N/A	N/A	**–**	**+**	**–**
Yadav et al., 2016	**+**	**–**	**–**	N/A	N/A	**–**	N/A	**–**	**–**	**–**	**+**	**–**	N/A	N/A	**–**	N/A	**–**	**+**	**+**
Papachristou et al., 2010	**–**	**+**	**–**	N/A	N/A	**–**	**–**	**–**	**–**	**–**	**+**	**–**	N/A	N/A	**–**	**–**	**–**	**+**	**+**
Huang et al., 2010	**–**	**–**	N/A	N/A	N/A	N/A	N/A	**–**	**–**	**–**	**+**	N/A	N/A	N/A	N/A	N/A	**–**	**–**	**+**
Fei et al., 2018	**–**	**?**	**–**	N/A	N/A	**–**	**–**	**–**	**–**	**–**	**+**	**–**	N/A	N/A	**–**	**–**	**–**	**?**	**+**
Kumar et al., 2017	**–**	**?**	N/A	N/A	**–**	**–**	N/A	**?**	**–**	**–**	**–**	N/A	N/A	**–**	**–**	N/A	**–**	**?**	**–**

*red, high risk of bias or concern; yellow, unclear risk of bias or concern; green, low risk of bias or concern; +, high risk of bias or concern; ?, unclear risk of bias or concern; −, low risk of bias or concern*.

## Discussion

### Summary of Main Findings

Severe acute pancreatitis is a serious state with high mortality and it requires high costs of the health care system. By more accurate prediction of the severity on admission, the risk of mortality can be reduced with the immediate optimal therapy.

Acute pancreatitis is diagnosed on the basis of the presence of two or more of the following three criteria: abdominal pain consistent with the diagnosis elevated pancreatic enzymes to a level of more than three times the upper normal value, and characteristic findings on abdominal imaging. Different radiological modalities (ultrasound, CT) are not only necessary to make the diagnosis of AP, but by the visualization of the gallbladder and biliary tract, they can reveal its etiology as biliary or non-biliary. Furthermore, by using morphological scoring systems e.g., CTSI or mCTSI, obtaining a CT scan can be useful for assessing the severity of AP.

This is the first meta-analysis, which quantifies the accuracy of CTSI and mCTSI scores for the prediction of the severity and mortality of AP, and compares them with other commonly used scoring systems. Two previous meta-analyses (Gao et al., 2015; Yang and Li, 2016) assessed the predictive accuracy of the BISAP score, but these articles did not contain CTSI nor mCTSI. Yang and Li (2016) found that the pooled sensitivity and specificity of the BISAP for the prediction of SAP were 0.65 (CI: 0.54–0.74) and 0.84 (CI: 0.70–0.92), respectively, the pooled AUC was 0.77 (CI: 0.73–0.80). Gao et al. (2015) calculated the pooled sensitivity as 0.51 (CI: 0.43–0.60), the specificity as 0.91 (CI: 0.89–0.92), the AUC was 0.87. Based on our results, we calculated 0.73 (CI: 0.53–0.87) for sensitivity, 0.80 (CI: 0.72–0.88) for specificity, and our pooled AUC was 0.79. The results are similar, the difference in specificity between our results and those of Gao et al. (2015) may be explained by the higher numbers of articles included in our analysis.

In our meta-analysis, APACHE II proved to be the most accurate scoring system for the prediction of mortality. It is the most widely used mortality prediction score in critically ill patients, however, it contains 12 points, including numerous clinical parameters, hence its application can be cumbersome and it limits its widespread use. In addition, APACHE II is designed for patients admitted to the intensive care unit, therefore it is not suitable for the early prediction of severity of AP. The AUC's of BISAP, mCTSI, and Ranson scores overlapped with APACHE II, while those of CTSI and CRP were mildly weaker.

Computed tomography severity index is accurate to predict severity, and its accuracy did not differ from the other scoring systems. However, the Ranson, APACHE II, and BISAP scores include several clinical parameters. There is a good correlation between morphological severity according to CT scoring systems and clinical scoring systems using clinical data and laboratory parameters.

The most recent guidelines of AP recommend a CT scan 72–96 h after the onset of the symptoms (Working Group IAP/APA Acute Pancreatitis Guidelines., 2013; Hritz et al., 2015), because pancreatic parenchymal necrosis in contrast-enhanced CT rarely appears within 48 h (Ryu, 2009). The guidelines allow an earlier CT scan in case of diagnostic uncertainty.

However, the contrast-enhanced CT examination cannot always be performed in every patient. In extreme obese patients, body weight, and size preclude the CT investigation. The contrast-enhanced CT assessment requires an intravenous injection of iodinated contrast medium for the detection of hypoperfused areas in pancreas parenchyma, therefore intravenous contrast media allergy, impaired renal function, and hyperthyroidism are contraindications.

Because of the risk of radiation exposure, repeated CT scans should be avoided, and should be reserved for patients who fail to improve clinically. CT examination had shown an advantage in evaluation of local complications (Ju et al., 2006), which can modify therapeutical strategy.

Contrary to the evaluation of Ranson, BISAP, and APACHE II scores, contrast-enhanced CT assessment, and CTSI calculation require radiological expertise.

### Limitations

Three of the articles contained data of children (Fabre et al., 2012; Lautz et al., 2012; Hashimoto et al., 2016), in 2 from these articles the DeBanto score was used for evaluating the severity of AP, which is a specific score for pediatric pancreatitis. The AP population of the studies is not necessarily representative for the whole AP population, because the CT scan is mostly performed in the more severe cases. In several studies not all etiologies of AP were included, Yang et al. included only patients with hyperlipidemic etiology while Alper et his coworkers included only biliary AP cases. Fifteen of the included studies were retrospectively designed, and these might have caused selection bias. The time of CTSI and mCTSI was not the same in the studies, in several studies it included a longer delay, while in others it was carried out on admission, leading to higher heterogeneity. While APACHE II, Ranson, BISAP and CRP values were established mostly on admission or within the first 48 h, the optimal timing of the CT examination 72–96 h after the onset of the symptoms and in several studies it was performed later than the other prognostic scores. This can limitate the prognostic score of the CTSI because the other scores can predict earlier severity or mortality with similar accuracy. It is also a good question if the radiologist can judge the time point of necrosis development. In populations with previous necrotizing pancreatitis, the severity cannot be accurately assessed.

For the value of predicting mortality, a considerable heterogeneity for CTSI and a substantial heterogeneity for Ranson score can be observed, while for the severity predicting value of AP a considerable heterogeneity for CTSI, BISAP, CRP, and Ranson scores and substantial heterogeneity for mCTSI can be noticed. We suspect that the confounder factors, that cause high heterogeneity among the studies are because of different population in terms of ethnicity, BMI, age (etc.), and etiology, the different timing and interpretation of imaging modalities, and potential inter observer variability between the radiologists interpreting the CT images. Because of the long delay characterizing the studies, the severity was assessed according to several Atlanta classifications and definitions.

## Conclusions

### Implications for Practice

In the prediction of mortality in AP, CTSI was revealed as equally valuable as BISAP, mCTSI, CRP, or Ranson score, only APACHE II score overcame its predicting ability. Considering severity, there was no difference in the prediction value of the scores. If CT scans are performed, CTSI and mCTSI can be easily calculated and should be used in addition to the other scoring systems.

### Implications for Research

Further research is warranted for the assessment of the effect of early CT and its predictive value in AP.

## Data Availability

The datasets generated for this study are available on request to the corresponding author.

## Author Contributions

ÉV and PH designed the research and the study concept. ÉV and AM performed the acquisition of data. PM and AS analyzed and interpreted the data. ÉV, AM, PM, AS, and PH wrote the article. AG, MB, BE, and LC supervised the study. BM, BE, JT, PS, and IR made a critical revision of the manuscript for important intellectual content. All of the authors gave their final approval of the version of the article to be published.

### Conflict of Interest Statement

The authors declare that the research was conducted in the absence of any commercial or financial relationships that could be construed as a potential conflict of interest.

## Footnotes

^1^https://www.stata-journal.com/sjpdf.html?articlenum=st0163
